# A Survey of the Influence of Process Parameters on Mechanical Properties of Fused Deposition Modeling Parts

**DOI:** 10.3390/mi13040553

**Published:** 2022-03-30

**Authors:** Ge Gao, Fan Xu, Jiangmin Xu, Guanghai Tang, Zhenyu Liu

**Affiliations:** 1 College of Mechanical Engineering, Jiangsu University of Science and Technology, Zhenjiang 212100, China; xufan@stu.just.edu.cn (F.X.); xujiangmin@just.edu.cn (J.X.); tangguanghai@just.edu.cn (G.T.); 2 Changchun Institute of Optics, Fine Mechanics and Physics, Chinese Academy of Sciences, Changchun 130033, China; liuzy@ciomp.ac.cn

**Keywords:** additive manufacturing, process parameters, fused deposition modeling, mechanical properties

## Abstract

Due to the availability of materials and low cost for production, fused deposition modeling is becoming the most widely used additive manufacturing (AM) technology. However, the reasonable choice of process parameters for FDM is a significant task that directly affects the performance of the printed part. Therefore, it is necessary to investigate the influences of various process parameters on the quality characteristics of the components. The objectives of this study are to thoroughly review the current state of research that characterizes, estimates the effects of process parameters on mechanical properties, and summarizes the conclusions of existing works. In addition, some general issues of the presented research are summarized, and the need for future development is also emphasized. Finally, the research proposes several areas that deserve further study in this field.

## 1. Introduction

Fused Deposition Modeling (FDM), also known as Fused Filament Fabrication (FFF), melts thermoplastic filament through a heater and deposits it layer by layer on the platform via a nozzle to form a part. The most significant advantage of FDM is the wide range of molding materials, which includes thermoplastic polymers in general. Sometimes low melting point metals, ceramics, and others materials are also used [1]. Besides, high speed, low cost, pollution-free, and simplicity of the process are also benefits of FDM. Consequently, FDM is emerging as the most widely used and embraced technique of additive manufacturing, which is applied in various fields such as aerospace, automotive, medical, and architecture with rapid growth [2]. However, anisotropic behavior, poor surface quality, and low dimension accuracy are drawbacks of FDM, usually resulting in poor mechanical characteristics of printed components, which dramatically limits the further application of FDM on a large-scale [3]. 

FDM is a complex process that has a large number of parameters that play different roles in the fabrication. To produce products with good quality and meet requirements for material behavior, it is necessary to evaluate the impact of these parameters on the characteristics. To date, many studies have been conducted to analyze different controllable parameters to achieve desirable properties of parts, including surface roughness [4,5], dimension accuracy [6,7], hardness [8], build time [9,10], and mechanical properties [11,12,13]. Obviously, mechanical properties are the most fundamental characteristics of FDM printed parts, among which tensile, compressive, and flexural strength are the three most important and concerning properties to the manufacturers and users, which are also the objects of this paper.

FDM involves various parameters that can be classified into three main types: process parameters(raster angle, layer thickness, build orientation, raster width, print speed, infill density, air gap, infill pattern, extrusion temperature); environmental parameters(platform temperature, envelope temperature, humidity, oxygen, etc.); other print parameters(nozzle diameter, material color, filament diameter, etc.). Although these parameters all affect the quality of FDM components, the contributions of which are different. Process parameters are the most commonly analyzed owing to their significant impact on mechanical performance and production efficiency. Actually, several published review papers related to the FDM process parameter are available for interested readers: Gordelier et al. [2], Dey and Yodo [14], Cuan-Urquizo et al. [15], Sheoran et al. [16], Mohamed et al. [17], Popescu et al. [18], Bakır et al. [19], Syrlybayev et al. [20]. These existing literature reviews generally investigate and analyze which process parameters can affect a certain material behavior. Since users of the 3D printer are directly faced with each process parameter, it is necessary and helpful to make them understand how each process parameter affects the quality and characteristics of printed parts at different values. However, to date, no literature review has been reported to explain the influence from the perspective of parameters rather than properties. As a complement, this survey focuses on functions of every process parameter with varying values and discusses the mechanism behind it by amalgamating collusions of existing studies from 2010 to 2021. Some research beyond this range is also included for important topics. This article aims to provide a comprehensive review of the roles of different process parameters in the FDM process, update the recent advances in process parameters optimization for researchers, serve as a resource for newcomers in this field and give directions for anyone wishing to improve the mechanical behaviors of their printed components.

The structure of the paper is organized as follows: Section 2 describes different process parameters and reviews literature related to investigating or improving the mechanical performance of FDM parts. Section 3 contains some key findings of the presented works and highlights concluding results. Section 4 describes difficulties encountered in the improvement of the FDM part characteristics. The last section includes recommendations and perceptions for future work.

## 2. Process Parameters

**Figure 1:**
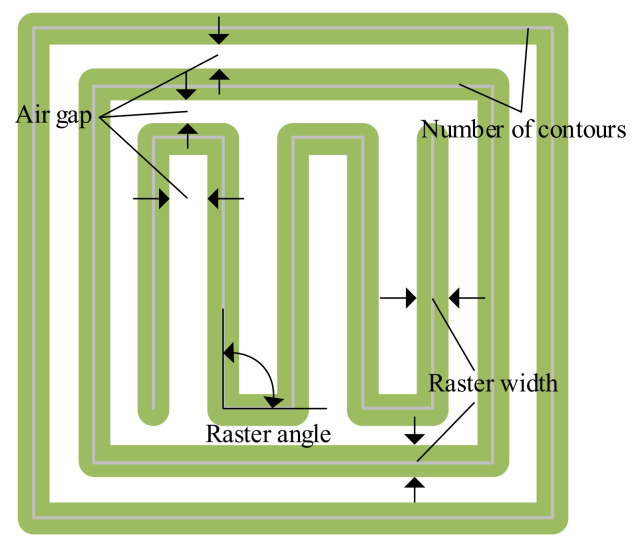
FDM process parameters related to toolpath.

The most researched process parameters include air gap, build orientation, extrusion temperature, infill density, infill pattern, layer thickness, raster width, raster angle, and print speed, as shown in Figure 1, which have substantial effects on filament (inter-layer and intra-layer) bonding, and thus influence the mechanical performance of FDM printed components [18]. In addition, interactions of these parameters play a significant role from the perspective of mechanical properties [21,22].

### 2.1. Build Orientation

**Figure 2:**
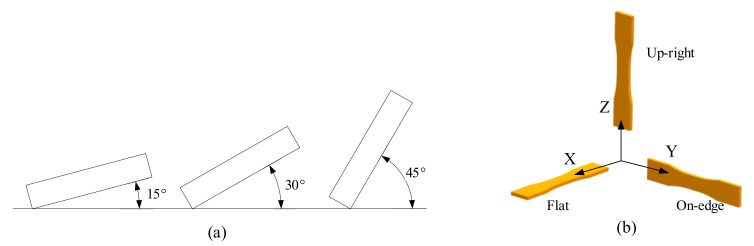
Build orientation: (**a**) arbitrary angle (**b**) certain angle.

Build orientation (or part orientation [23], construction/layer orientation [24]) represents how and in which direction the part is generated on the print platform. In fact, build orientation can represent an arbitrary angle with any value [8,25,26], but in most studies, it is regarded as a certain angle with respect to X, Y, and Z-axis [27,28]. Generally, when test specimens are placed horizontally, vertically, and laterally, the build orientation is named as flat, upright, and on-edge, respectively, which is shown in Figure 2. Flat and on-edge are considered parallel to the print platform, while upright is along the direction of normal of the print platform.

The influences of build orientation on the mechanical performance of FDM components have been extensively researched. Different authors investigated the relationship between various materials and building directions. Wang et al. [7] established building factors with various levels based on analysis of variance (ANOVA). The result verified build orientation in the Z-direction to be the most predominant factor for tensile strength. Lee et al. [29] showed that compressive strength of ABS parts was maximum at 0° build orientation. Gorski et.al. [30] noted that tensile strength was maximum at 0° for ABS filaments. Moreover, they found the specimen presented brittle behavior instead of ductile behavior as build orientation increased exceeding certain angles. The conclusions were consistent with Ashtankar et al. [25]. Their study reported that tensile strength of ABS specimen decreased, with the increase of build orientation from 0° to 90°. This trend was also applicable to ultimate compressive strength, which was minimum at 90° orientation. In another study, Hernandez et al. [31] experimentally determined both compressive properties and flexural properties were maximum at 0° build orientation for ABS P430 filaments. Besides, compressive strength was minimum at 45° build orientation. They also deduced that the effect of build orientation on tensile strength of ABS printed parts was insignificant. Bertoldi et al. [32] and Zou et al. [33] experimentally showed that build orientation strongly affected tensile strength and elastic modulus, respectively. Raney et al. [34] evaluated the effects of build orientation and infill density on tensile strength of ABS parts manufactured by a uPrint SE 3D printer, showing that the strength of samples tested against the layers was less than 80% of that tested along the layers. 

As for materials other than ABS, Domingo-Espin et al. [35] tested tensile strength of PC parts. This group of researchers proved that tensile strength was maximum at 0° build orientation. Smith and Dean [36] also pointed out that, compared to bulk material, there was a 45 percent decrease in elastic modulus and a 30 to 60 percent decrease in ultimate tensile strength of PC parts depending on orientation. Zaldivar et al. [37] revealed that FDM materials behaved more as laminated composites with macrostructures than isotropic cast resins, consequently tensile strength, failure strain, Poisson’s ratio, coefficient of thermal expansion, and modulus all varied significantly depending on the build orientation of PEI dogbones. Taylor et al. [38] analyzed the flexural behavior of PEI parts with varying build orientation and raster angle experimentally and numerically. Results indicated that modulus and yield strength were influenced by an interaction between these two parameters.

In summary, build orientation significantly affected the mechanical properties, which usually played the predominant role when compared to other parameters [39]. For arbitrary angles, in case of other parameters such as air gap and raster angle are kept constant, the 0° orientation is preferable, which shows the highest values for maximum tensile strength, compressive strength, and flexural strength. Consequently, flat or on-edge oriented samples usually exhibit inter-layer failure with higher stiffness and strength performance. On the other side, increasing the angle from the build platform results in microstructures that further reduce the volume fraction of extruded fiber material from the primary load direction resulting in lower strength. That is why upright samples showed inter-layer failure with lower stiffness and strength performance.

### 2.2. Raster Angle

**Figure 3:**
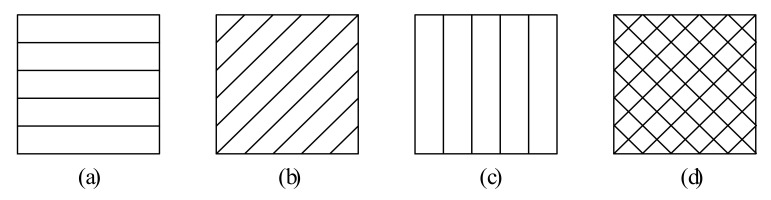
Raster angle: (**a**) 0° (**b**) 45° (**c**) 90° (**d**) −45°/45°.

Raster angle (sometimes called raster orientation [40], layer orientation [41], fiber orientation [42], or even pattern orientation [43]) represents the angle of the filament direction with regard to the X-axis (usually load direction) of the platform. The allowed raster angles can vary from −90° to 90°, and typically used values are 0° (axial), 45°(cross), 90° (transverse), and their combination. For example, −45°/45°(criss-cross) represents the raster printing directions are −45° and 45° alternately for different layers, as shown in Figure 3.

Ahn et al. [44] applied the Tsai-Wu failure criterion and classical lamination theory to reasonably predict the anisotropic failure model for FDM parts as a function of raster angle. Magalhães et al. [45] suggested that proper choice of raster angles in sandwich specimens could gain in the strength and stiffness of parts, compared to default (45°) FDM configuration. Ziemian et al. [46] and Zhou et al. [47] indicated that the highest tensile strength was obtained at raster angle with 0° for ABS and PP-PC composites, respectively, while the specimens with 90° raster angle exhibited the minimum strength. Es-Said et al. [40] and Garg et al. [48] drove a similar conclusion for flexural strength as well as tensile strength. Moreover, Ziemian et al. [49] further reported that 45° raster specimens in compression were significantly weaker than other raster angles. Based on the analysis of biaxial raster angles, Fatimatuzahraa et al. [50] noted that the structure of 45°/−45°provided better flexural strength than that of 0°/90° of ABS built specimens, despite the almost equivalent tensile strength [51]. A similar conclusion for tensile strength was also driven by Diaconescu et al. [52]. Hart and Wetzel [53] explored the fracture properties of ABS parts with different raster angles. Results confirmed that the elastic-plastic response of the material depended on the raster angle of printed specimens. In contrast, Arbeiter et al. [54] reported that fracture behavior might be not highly dependent on the raster angle by setting ideal processing parameters of PLA samples.

The interaction of build orientation and raster angle can cause strong anisotropy of the FDM parts, therefore these two parameters are generally studied together. Rohde et al. [12] revealed that ABS and PC samples exhibited strong anisotropy as functions of build orientation and raster angle, respectively. Shear moduli were affected by build orientation rather than raster angle for ABS specimens. The lowest values of modulus of rigidity, ultimate shear strength, and yield shear strength were obtained from on-edge configuration specimens. Durgun and Ertan [23] reported that the build orientation had a more significant influence than the raster angle on the mechanical behavior of the resulting fused deposition part. Small raster (e.g., 0° angle) resulted in increased strength resistance in all component positions. Bellini and Güçeri [55] carried out analytical and experimental approaches to study the effect of build orientation and raster angle on flexural strength and tensile strength of ABS material. Balderrama-Armendariz et al. [56] studied elastic properties in torsion of ABS-M30 samples at different build orientations and raster angles. They characterized that build orientation had an insignificant modification of the response of 0.2% yield strength or ultimate shear strength, while the orientation in YXZ with raster at 0° led to improved responses in all measured torsion parameters. Cantrell et al. [57] showed that build orientation and raster angle had a negligible influence on the tensile modulus of ABS specimens. The highest tensile properties and highest shear strength were found in specimens with on-edge orientation and specimens with [+45°/−45°] flat orientation, respectively for PC material. In addition, the shear modulus was almost the same for all specimens with [+45/−45] raster angle regardless of build orientation. Torrado et al. [58] explored the effects of build orientation and raster angle on mechanical anisotropy. The tensile test results exhibited an equivalency between different sample types. Therefore, the authors recommended horizontal specimens printed with a transversal filling due to its higher reliability, higher accuracy, and simplicity of the printing process. Letcher et al. [59] investigated the relationship between layer number, raster angle, and mechanical properties of ABS printed specimens. Results showed that 0° raster orientation yielded the highest strength at each layer number. Furthermore, maximum stress and elastic modulus increased with the increase of the number of layers.

In summary, the relative position of fibers and the axial load causes the specimens to react differently. Raster angles with a higher fraction of specimens oriented along the axis of the load (e.g., 0° orientation) exhibit improved tensile and compressive strength of the part, while those that are offset (e.g., 90° orientation) exhibit reductions in mechanical performance [60,61,62,63]. In the former case, fibers themselves withstood most of the applied load, resulting in inter-layer failure. While for the latter case, bonding between adjacent layers and rasters withstood the load, resulting in trans-layer failure, which is much weaker. A similar trend is applicable to the flexural specimen, which can be regarded as one side experiencing compression while the other side experiencing tension when loaded.

### 2.3. Layer Thickness

**Figure 4:**
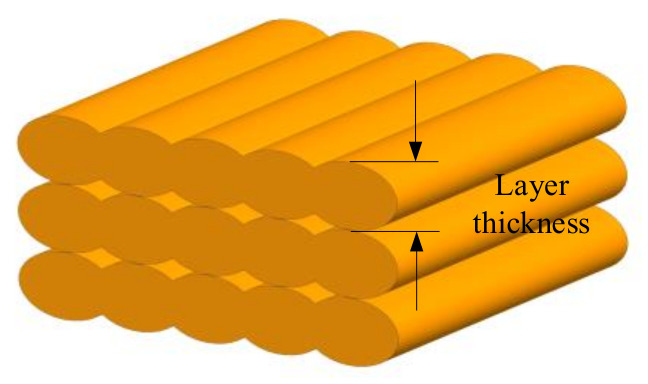
Layer thickness.

Layer thickness (or layer height [64]) represents the thickness of the layer printed by the nozzle tip, as shown in Figure 4. In general, it is smaller than the diameter of the extrusion nozzle (usually one-half), depending on the material and tip size. Layer thickness is directly related to the number of layers printed and hence print time. It has been verified that better accuracy of the component can be achieved by setting lower layer thickness.

Layer thickness is usually studied together with other parameters, most commonly with raster angle. Somireddy et al. [42] researched the influences of raster angle and layer thickness on the flexural behavior using classical laminate theory. Results presented that thinner layer laminates have higher loading capacity and flexural stiffness than thicker ones, except for the maximum deflection. Tymrak et al. [63] quantified the elastic modulus and tensile strength of PLA and ABS parts by comparing different layer thicknesses and bidirectional raster angles. Tests showed that tensile strength dropped with increasing layer thickness. In another study by Rankouhi et al. [62], the mechanical characterization of PLA by varying layer thicknesses and raster angles were analyzed. The maximum elastic modulus and ultimate tensile strength were obtained at lower values of both two factors. Similar results can be obtained for other materials, such as PEEK (Wu et al. [65]) and plaster-based powder (Vaezi and Chua [43]). Garg and Bhattacharya [66] considered layers of different thicknesses and rasters at different angles by simulation and experiment. FE analysis indicated that tensile strength, strain at yield, elongation, and developed stress first decreased with an increase in layer thickness and then increased. Layer thickness, build orientation, and raster angle were evaluated parameters to examine their effects on tensile strength by Nidagundi et al. [67]. Thinner layer thickness, 0° build orientation, and 0° raster angle were optimum for ultimate tensile strength.

In comparison, Rodríguez et al. [24] compared the effect of build orientation, infill density, and layer thickness on the mechanical characteristics of ABS and PLA test components. Regarding ABS, the mechanical strength results barely varied with respect to layer thickness. In contrast, tensile strength of PLA decreased as layer thickness increased. Chacón et al. [27] characterized the effect of layer thickness, build orientation, and print speed to determine the mechanical response of the PLA specimens. They observed that the increased print speed and layer thickness caused ductility to diminish. In addition, the mechanical properties for the upright orientation increased as layer thickness increased and as the print speed decreased, which however were of slight significance for on-edge and flat orientations. Alafaghani et al. [28] demonstrated that mechanical properties were significantly influenced by build orientation, extrusion temperature, and layer thickness; and less significantly on infill pattern, for high infill density specimens, and print speed. To improve the mechanical properties, higher extrusion temperature and larger layer thickness are needed in addition to appropriate build orientation. Carneiro et al. [68] mechanically assessed the influence of raster angle, layer thickness, infill density of PP and GRPP composites. The results showed the infill density had a linear effect on both mechanical properties. Instead, layer thickness had an insignificant effect on the performance of samples. Dong et al. [69] demonstrated that the number of layers was the only dominant factor in improving mechanical strengths of PLA and PLA/wood composites, compared with infill density and layer thickness.

In summary, layer thickness has a different effect on the strength. For a given total height, the thickness of a layer has an inverse proportional relationship with the number of layers. The thinner the layer thickness, the more layers. This response will lead to a high-temperature gradient towards the bottom of the component, which will improve diffusion between adjacent rasters, thus ultimately contributing to the load-bearing and enhancing the strength. In addition, this trend is heightened when at low print speed, which gives a better bonding with the previous layer. On the other hand, an increase in the number of layers also adds to the number of cooling and heating cycles, which in turn gives rise to residual stress accumulation. This behavior can result in distortion and inter-layer cracking, which will reduce the strength. Due to the interaction of these two different influences, in general, a moderate thickness value is obtained as the optimal parameter in some research [70].

### 2.4. Air Gap

**Figure 5:**
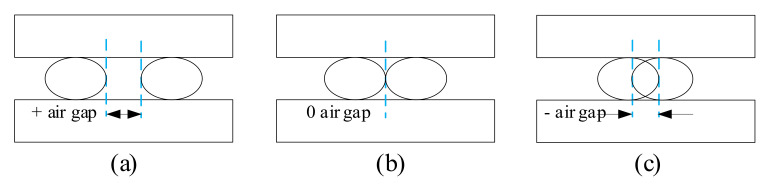
Air gap: (**a**) positive air gap (**b**) zero air gap (**c**) negative air gap.

The air gap represents the space between two neighboring printed filaments on the deposited layer. In most cases, the air gap represents the distance between rasters, viz. raster to raster air gap. However, in some research, the air gap is distinguished as raster to contour air gap and contour to contour air gap, respectively. In general, there are three types of air gap, and they are zero, positive and negative. The zero type is generally the default configuration, which places beads just alongside each other. The positive type has a loose place between beads which results in rapid building, while the negative type means that two beads partially overlap the structure, creating a denser component, as shown in Figure 5.

Rodriguez et al. [71] observed three monofilaments with different air gaps made of ABS. From all arrangements tested, the highest stiffness and tensile strength values were found for the filament with rasters aligned in the loading direction and a small negative air gap. Too et al. [72] characterized that the air gap size had a profound impact on the porosity and compressive strength of FDM built part. With the increasing air gap of the test specimen, compressive strength decreased while porosity increased, respectively. Dawoud et al. [73] researched the impact, flexural and tensile strength of ABS components with different raster angles and air gaps. The air gap with a negative value proved to be the most significant factor in the enhancement of mechanical properties. However, in the case of a positive air gap, varying raster angles seemed to have a more significant effect on tensile strength. Masood et al. [74] presented experimental work on the effect of raster angle, raster width, and air gap on tensile properties of PC. They reported that the air gap was the only dominant parameter influencing tensile properties. This study also found that PC material by FDM had tensile strength in the range of 70 to 80% of the injection molded and extruded PC parts. 

In the study of Slonov et al. [75], raster angle, air gap, and raster width on the mechanical properties of samples from PPSF were examined. The authors found that the elastic modulus generally depended on the air gap between rasters, independent of raster angle. On the contrary, the impact strength depended on the raster angle and the adhesion degree between filaments. Hossain et al. [76,77] modified raster width, raster angle, raster to raster air gap, and contour width to improve tensile mechanical properties of PEI material by visual feedback method. Using negative raster to raster air gap led to an average increase in ultimate tensile strength of 16%, compared to the default configuration. Montero et al. [78] examined five process parameters (raster angle, raster width, extrusion temperature, air gap, and color) to understand the ABS properties fabricated by FDM. They observed that the raster angle and air gap influenced tensile strength FDM printed part, while color, extrusion temperature, and raster width had little influence. Moreover, stiffness and shear strength between roads were lower than those measured between layers. Bagsik and Schöppner [79] considered the effect of build orientation, air gap, raster angle, and raster width based on the mechanical data of PEI. Based on their study, the air gap with a negative value contributed to the best results for all directions. With thicker filaments, better mechanical performance could be obtained for the on-edge and upright build direction, while a thinner filament enhanced the strength properties of the flat specimens. 

In summary, air gap determines the area of force bearing as well as bonding between filaments. From the perspective of effect, the work of the former one on the mechanical property is more apparent than that of the latter one. In general, the positive air gap results in a loosely packed structure with weak bonding between adjacent filaments, leading to lower strength. In contrast, the negative air gap results in a denser squeezed structure with strong interfacial bonding, significantly improving the strength. Zero air gap may enhance the diffusion between the neighboring rasters, and cause the total bonding area to diminish as well.

### 2.5. Raster Width

Raster width represents the width of the printed beads or roads for rasters. It depends on the nozzle tip size. Some researchers distinguish contour width from raster width [80,81], as shown in Figure 1. However, in most studies, contour width and raster width are regarded as the same parameter, represented by road width [11,65,82].

Gebisa and Lemu [80] focused on processing parameters, such as contour width, raster angle, contour number, raster width, and air gap, on the effect on the flexural properties of PEI-manufactured parts, which could be arranged as importance: raster width and raster angle > contour width and contour number > air gap. They also found that the effect of a minus air gap could differ between two different materials, which was not recommended for PEI. Ang et al. [83] specified process parameters, namely air gap, raster width, build orientation, number of layers, and infill pattern, on the compressive properties and porosity of ABS scaffold structures. The experiment determined raster width and air gap as the most significant parameters. Moreover, porosity decreased when the air gap decreased or raster width increased. In contrast, compressive strength and modulus increased as raster width increased while the air gap decreased. Rayegani and Onwubolu [84] used the group method of data handling (GMDH) and differential evolution (DE) to quantify the effects of air gap, raster angle, build orientation, and raster width on tensile strength. The investigation showed that negative air gap, as well as smaller raster width, significantly improved tensile strength. Particularly, build orientation played a major role, as could be observed from the results. Onwubolu et al. [85] applied the design of experiment (DOE) to study the main and interaction effects of process variables such as build orientation, raster width, layer thickness, air gap, and raster angle on tensile and strength of ABS components. The maximum tensile strength was obtained with zero build orientation, maximum raster width, raster angle, and negative air gap. In Liu et al. [86], five input process parameters such as build orientation, layer thickness, raster orientation, air gap, and raster width were considered to examine their influence on impact, flexural and tensile strengths. The optimum combination was obtained based on analysis of variance and gray relation analysis. Gkartzou et al. [87] examined the influence of raster width on tensile properties of PLA/Lignin composites. The results showed that specimens with different raster widths had similar tensile strength and Young’s modulus. 

In summary, larger raster width creates a high temperature near the boding surfaces and a larger bonding area, which may improve the diffusion and lead to stronger bond formation [64]. However, a larger raster can also result in stress accumulation along the width of the part, as well as deterioration in thermal conductivity [88]. On the other hand, smaller raster width will require less production time and material. On the whole, at the intermediate value of the raster width, the higher thermal mass that cools slowly can be achieved, which enhances the bonding between the filaments and thus improves the strength [89].

### 2.6. Infill Density

The outer region of AM part is usually solid, but the interior area, generally known as the infill, is the inner component covered by the skin, which has different geometries and sizes. Infill density (or infill degree [68], infill ratio [82], infill percentage [90], fill density [88]) refers to the percentage of filament material printed in the given part, where 0% is a shell and 100% is a solid. FDM technology allows users to control the infill density through parameters such as air gap or raster width. 

Alvarez et al. [90] observed that the maximum impact resistance, tensile stress, and tensile force were obtained with 100% infill density. Martikka et al. [91] revealed that the increment in infill density enhanced the tensile properties of PLA and PLA/wood composites. Gomez-Gras et al. [92] carried out the Taguchi method to investigate the impact of four process parameters and their intersections—layer thickness, infill density, nozzle diameter, and print speed, on fatigue response. It was concluded that infill density showed the strongest influence in fatigue performance, followed by nozzle diameter and layer thickness, whereas print speed showed no relevant effect in PLA specimens. Aw et al. [93] looked at relating process parameters to tensile properties of CABS/ZnO composites with infill density and infill pattern. Results revealed that tensile strength of CABS composites was little affected by the change of infill density, while the increased infill density caused Young’s modulus to increase, resulting in higher stiffness. Line pattern possessed better tensile properties. Kerekes et al. [94] pointed out that with an increase in infill density, Young’s modulus, initial yield stress, ultimate strength, and toughness increased, while elongation at break decreased. Layer thickness showed a moderate influence affecting the specimen’s properties, where an increasing layer thickness apparently increased Young’s modulus, while it decreased elongation at break. Lužanin et al. [95] experimentally analyzed flexural properties depending on the infill density, layer thickness, and raster angle. The researchers reported that layer thickness was the most important parameter affecting flexural force, and the interaction between infill density and raster angle was significant as well. The mechanical effect of printing parameters for carbon fiber-reinforced polyamide was studied by Toro et al. [13]. The most dominant parameter was found to be infill density. Layer thickness and infill pattern played importantly in flexural and tensile behaviors, respectively.

In summary, the mass and strength of FDM produced parts are dependent on the infill density. Lower density requires less print time and material, thus saving cost and reducing the weight. However, more voids are generated within the structure simultaneously, leading to increased porosity. As a result, the dimension of the bonded region between filaments decreases and so as well to the mechanical properties. In contrast, the denser component possesses better mechanical properties but takes much more time to be complete. For example, the specimen built with 100% infill density usually exhibits maximum strength. Generally, infill density ranging from 50% to 98% is recommended, since the improvement in mechanical resistance is countered by longer manufacturing times [90].

### 2.7. Infill Pattern

**Figure 6:**
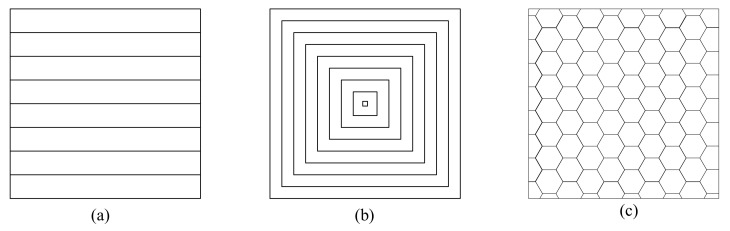
Infill pattern: (**a**) linear (**b**) concentric (**c**) hexagonal.

Infill pattern (or print pattern [93]) represents the way how filaments fill and cross the internal space of the printed part, as shown in Figure 6. Different infill patterns usually have different geometrical layouts and complexity, which will affect print time and the material used.

Many filling patterns are available such as hexagonal (or honeycomb), linear, and diamond, as illustrated by Alafaghani et al. [28], in which the commonly used is the hexagonal pattern. Cho et al. [96] compared the influence of PLA samples with different infill patterns and layer thickness on tensile property. They concluded that layer thickness had a higher effect than infill pattern, and the triangle pattern gave the highest mechanical strength and lowest material consumption. Dave et al. [97] investigated the effect of three process parameters: infill pattern, infill density, and build orientation, on the tensile properties of PLA specimens through a full factorial experiment. ANOVA results indicated that infill density was the most predominant process parameter for tensile strength, compared with infill pattern and build orientation. Fernandez-Vicente et al. [98] found that changes in infill density determined mainly tensile strength of ABS material. At the same time, the influence of the different infill patterns caused a variation of no more than 5% in maximum tensile strength, along with similar behaviors. Akhoundi et al. [99] identified the key factors that influenced tensile and flexural strengths. The input variables, such as infill pattern and infill density, and their relationship with raster angle and void presence, were considered. The result concluded that the highest tensile and flexural strengths were obtained by concentric pattern. They also found that when rasters were deposited at short distances in the Hilbert curve, a high temperature was maintained, which resulted in better fusion and strong bonding between the adjacent rasters. Baich et al. [9] presented the relationship between various infill patterns and different mechanical properties. Statistical analysis revealed that for double-dense infill in all loading conditions, solid infill showed higher strength at the same fabrication cost. Therefore, solid infill was recommended for mechanical applications, in the case of entry-level printers. Moreover, compressive strength increased as the complexity of the infill pattern increased. Nagendra and Prasad [100] revealed significantly linear interactions between infill pattern and other process parameters, such as extrusion temperature, layer thickness, and infill density, on mechanical properties of Nylon/Aramid composite.

In summary, the infill pattern has a complex effect on the mechanical properties of parts produced by FDM owing to a broad spectrum of types. For example, in the hexagonal pattern, each layer lays down on a similar previous layer, the same as the bonding zone. While in the rectilinear pattern, the lay crosses the previous layer at points, which correspond with the bonding zone between each layer. However, the combination of rectilinear patterns in a 100% infill shows higher tensile strength, compared with the honeycomb pattern [98]. Therefore, these results need to be analyzed and explained with caution.

### 2.8. Print Speed

The print speed (or feed rate [27], print velocity [92], infill speed [101], deposition velocity [102]) represents the speed of the nozzle traveling relative to the print platform. Generally speaking, the lower the print speed, the longer the production time and the better the accuracy of the prints. In comparison, the higher the print speed, the faster parts are produced.

Christiyana et al. [103] produced ABS composite specimens and investigated the role of print speed and layer thickness. It was observed that the maximum flexural and tensile strengths were achieved via setting thinner layer thickness and lower print speed. Similarly, Ning et al. [101] showed that tensile strength of CFRP composites decreased with the increase in print speed. Santana et al. [104] analyzed the factors affecting PLA parts with variations in print speed and extrusion temperature to evaluate the quality of the open-source 3D printer. Based on the value, the print speed and extrusion temperature were irrelevant compared with the mass and modulus of rupture. Kačergis et al. [105] investigated the influence of print speed, platform temperature, and number of layers in the structure printed with PLA and TPU. Experimental results proved that the deformation was strongly influenced by the print speed. By contrast, Li et al. [21] pointed out that air gap played a predominant part in determining tensile strength, followed by layer thickness, and the effect of print speed is the weakest factor. They suggested that smaller values of layer thickness and air gap were preferred if higher tensile strength was needed. Furthermore, print speed could be set relatively higher to improve fabrication efficiency. Lužanin et al. [106] studied the relationship between the maximum flexural force of PLA parts and five process parameters. The input variables were extrusion temperature, infill density, print speed, raster angle, and layer thickness. The optimal parameters setting was maximum levels of infill density and print speed, mid-level of layer thickness, and minimum level of raster angle.

In summary, the effect of print speed on mechanical performance shows a different trend. Generally, lower print speed gives a better bonding and interaction between contiguous filaments, leading to an increase in tensile and flexural strength. However, if the print speed is too slow, the too-long inter-layer cooling time makes just-deposited material cool down at a lower temperature, which disfavors the fusion of the thermoplastics, hence the strength and ductility are affected [107]. On the other hand, rapid print speed could improve the efficiency, but leave not enough time for extrusion materials to plasticize, and the amount of residual stress produced during deposition increases significantly as well [108], which leads to weak mechanical properties. It should be pointed out that the production time is not only affected by print speed but also related to build orientation. Print time decreases as print speed increases for on-edge and flat orientations, while print time remains almost constant for up-right orientation with high-speed values [27].

### 2.9. Number of Contours

The number of contours (or number of perimeters [109], number of shells [110]) refers to the number of closed roads that are deposited along the edge of the part, as shown in Figure 1. It may range from one to the number of filaments extruded.

Kung et al. [109] studied the influences of three process parameters including number of contours, raster angle, and specimen size. They pointed out that there existed apparent dispersion of the strength for a different number of contours. Interestingly, they also noted that tensile strength of specimens built with 45° is greater than those built with 0°. According to Mahmood et al. [110], there was a positive relationship between tensile strength and number of contours. In addition, a larger cross-section negatively affected tensile strength of a printed part while keeping the other parameters constant. Croccolo et al. [111] experimentally and analytically dealt with the effect of contouring on the static strength and stiffness of ABS parts. They showed that the larger the number of contours, the greater the elastic modulus and stiffness, and thus the higher the maximum strength. Moreover, with the increase of the number of contours, the percentage of elongation to failure decreased. Griffiths et al. [112] performed an experimental investigation on the tensile property of PLA objects. They utilized a full factorial DOE approach considering building orientation, infill density, number of contours, and layer thickness as parameters. The study concluded that the infill density and number of contours were the only significant parameters that should be maximized for optimization. Lanzotti et al. [61] observed the increase in strength with the number of contours and layer thickness. In particular, the strength increased as the raster angle decreased with a rate that was as greater as the layer thickness increased.

In summary, the number of contours impacts the mechanical properties of the part fabricated. When the number of contours increases, the effect is directly seen in the increase in strength. This is owing to the fact that the load is applied directly on the contour rather than the rasters, therefore a growing contour number causes the raster length and number of rasters to decrease, which will lead to improvement in the performance of the part.

### 2.10. Extrusion Temperature

Extrusion temperature (or print temperature [82], nozzle temperature [113]) refers to the temperature at which the fibers are heated inside the nozzle during the FDM process. It can influence the fluidity and solidification characteristics of the molten material and control the viscosity of filament extruded from the nozzle.

Deng et al. [82] applied an orthogonal test to evaluate the effects of process parameters such as print speed, layer thickness, extrusion temperature, and infill density, on tensile properties of PEEK components. They demonstrated that more micro-pores and slag inclusion were caused by lower print speed and extrusion temperature, leading to lower strength specimens. Aliheidari et al. [113] designed double cantilever beam specimens of ABS and printed at different extrusion temperatures to study the mode-I fracture resistance. Based on critical J-integral value, the authors stated that the higher the temperature was, the greater number of polymer molecules were inter-diffused at the interface, which resulted in higher resistance to fracture. Rinanto et al. [114] optimized extrusion temperature, infill density, and raster angle to produce prototypes with high tensile strength. The optimization combination was 45° of angle, 40% of density, and 210 °C of temperature. Among these three parameters, infill density is the most predominant factor. Sun et al. [115] explored the influence of extrusion temperature and envelope temperature on the quality of bonds between adjacent ABS filaments. Statistical analysis proved that both the envelope temperature and variations in the convective conditions within the printer have substantial influences on the mesostructure and the overall quality of the bond strength between rasters. Leite et al. [116] determined the influence of mechanical properties from layer thickness, extrusion temperature, raster angle, and infill density. The best values reported for the sample were higher infill density and extrusion temperature, and lower layer thickness. Sun et al. [117] demonstrated that increasing platform temperature could enhance the PEEK binding force between layers, making the model more excellent mechanical properties. Moreover, low infill density could also improve the performance of the material. Yang [118] observed a decrease in tensile and flexural properties of WFRPC components with an increase in the extrusion temperature, whose trend is opposite to that of compressive strength. 

In summary, the extrusion temperature has an important effect on the crystallinity of the material and polymer filament bonding. Thus, the mechanical performance of printed parts will be affected as well. Higher extrusion temperature of the deposited filament gives better inter-layer fusion, which results in higher mechanical properties. However, too high extrusion temperature may cause material degradation or molding failure during deposition, resulting in dimensional inaccuracy and filament deformation [82]. On the other hand, lower extrusion temperature may prevent the material from melting adequately, leading to nozzle clogging. Both of the two cases above will lead to weak mechanical properties of printed parts.

## 3. Results and Discussions

In an effort to aggregate thorough information on process parameters of the FDM technique and their influence on mechanical properties, we have summarized the research works in the field concisely. Table 1, Table 2, Table 3, Table 4, Table 5, Table 6, Table 7, Table 8, Table 9, Table 10 and Table 11 give an overview of the parameters and mechanical properties of FDM products intensively investigated in the literature. In most existing research, several parameters are studied together. Therefore, the parameter that plays a major role or authors of the research care about most as the basis for classification. For the case of many parameters included, we attribute it to Table 11 (Others). However, for certain process parameters, there is not much research. Therefore, all studies containing this parameter are grouped into its table. As a consequence, the criteria for the aggregation of these tables are not strictly unique. Since there is much scattered data and information, interested readers are encouraged to review the references provided according to their interests. The key findings of this survey are summarized below:

- The work of different process parameters is coupled and combined to affect the mechanical property of FDM parts, which all have importance and effects. Generally speaking, there exists a parameter playing a dominant role. For example, extrusion temperature, layer thickness, air gap, and print speed can influence the heat transition of the structure, thus affecting the bonding between rasters and the mechanical characteristics. However, extrusion temperature is the most significant factor in determining temperature field variation, followed by layer thickness, print speed, and air gap by order of importance [102,135].
- One process parameter may affect or be affected by several other parameters, directly or indirectly. For instance, layer thickness affects the raster width and print speed. Likewise, the number of layers is related to build orientation and layer thickness in a part. What is more, infill density values significantly have an impact on the print speed, which can be changed by adjusting air gaps and raster width.
- The contribution of a single parameter may be contradictory from different aspects, which should be determined by the final effect. A typical example is raster angle. Small raster angles (e.g., 0°) will contribute to load-bearing due to filament lying along the loading direction. On the other hand, they will also lead to long rasters, which result in stress accumulation and hence weak bonding [22]. However, the final effect is that a small raster angle ensures the best tensile, compressive and flexural strength, proving that the former one plays a dominant role.
- Optimal parameter values obtained are just in theory, which should be reconsidered and adjusted in practice. According to the conclusion obtained in the former section, thinner layer thickness can help reinforce the tensile strength of the part, which, however, costs more due to more material and time usage for producing [136,137]. Consequently, a compromise needs to be made between improving property and reducing cost.

## 4. Research Shortcomings and Challenges

This paper reviewed the literature concerned with the effects of various process parameters on mechanical performance by investigating their individual/combined effect. Despite the achievements of the current work, this section describes the major challenges and shortcomings of recent research.

### 4.1. Diversity of Materials

In most presented research, influences of materials and printers are neglected insignificantly, in fact. From tables, it can be seen that there is a variety of materials for FDM, among which ABS and PLA are the two most widely studied. Other few known materials such as PC [35,57], PEI [37,79], PEEK [138], and Nylon [139] occupy only a small part of the research, not to mention PP [68], PPSF [75], PETG [140,141], or composite materials [93,142]. Therefore, conclusions about process parameters of most studies are obtained from ABS and PLA, which may be not applicable to other materials. For example, negative air gaps are preferred to enhance tensile and flexural behavior for ABS, as demonstrated by multiple works [11,73]. However, for structural materials such as PEI, a minus air gap is not recommended. As this material is processed at high temperature, and zero air gap is sufficient to improve mechanical properties flexural strength by adjusting other parameters, which can reduce the loss of dimensional accuracy and surface quality, caused by the usage of a negative air gap [80]. It should also be noted that materials from different suppliers differ in quality [141]. Moreover, even though the same material from the same source in different colors can lead to variation in properties. For instance, Wittbrodt et al. [143] reported that colors influenced the crystallinity percentage of polymers, and thus impacted the strength, which could not be deemed a low level of significance [44,121]. Therefore, research in a wider variety of materials will contribute to understanding the effect of process parameters better and help overcome shortcomings of FDM.

### 4.2. Variety of Printers

There exist a wide range of machines from different manufacturers, as presented in the tables. Although samples are from the same material, they may have different properties when printed by other printers [144]. For instance, Tymrak et al. [63] found that ABS parts in a 0° orientation had elastic moduli around 1900 MPa and tensile strengths nearing 30 MPa by RepRap printer, which was higher than that in similar studies from different commercial printers, with moduli varying between 1000 and 1700 MPa, and tensile strengths ranging from 10 to 18 MPa [55,71]. The influence of 3D printers on the mechanical property of FDM parts is definite and obvious. However, there is still a lack of adequate and specific means to measure or evaluate this impact. An effort should be made to identify standard and test methods that could be used to validate FDM machine performance.

### 4.3. Difference in Results

Since FDM is a complex process, it is difficult to replicate the experiment completely from others, which may lead to different or even opposite conclusions. For example, Dawoud et al. [73] showed that an air gap with a negative value could improve the mechanical property. On the contrary, Mohamed et al. [17] claimed that a positive air gap facilitated the spread of semi-molten materials between the gaps, which led to stronger structures. This phenomenon is more apparent when it involves multi-parameter optimization. As another example, Panda et al. [26,133] investigated process parameters (air gap, build orientation, raster angle, layer thickness, raster width) for mechanical properties of ABS parts. Experiments were conducted using a central composite design and part swarm optimization, respectively. However, the optimum process parameters obtained were different from that by Rayegani et al. [84]. In a word, samples with the optimal combination of parameters may have similar strength to those under the opposite parameters setting. That is why it is difficult to evaluate the role of a specific parameter in a multi-parameter combination.

### 4.4. Limitation of Research Parameters

It is clear that some of the process parameters are widely studied: infill density, layer thickness, raster angle, build orientation, and air gap. Print speed and raster width also occupy a place in the research field. However, other parameters such as infill pattern, number of contours and extrusion temperature are the least analyzed, which needs more attention. For example, the road width for raster and contour is assumed to have a similar effect on the properties in different studies. However, Gebisa and Lemu [80] concluded that raster width and contour width were two different parameters with completely different influences, which needed to be examined separately. For another instance, raster angle 0° ensures the best mechanical strength, presented by many researchers, while Dave et al. [97] found that samples built with raster angle 90° in Hilbert curve pattern displayed a better result as compared to 0° value. These different results indicate that researchers should spend more time investigating the “ignored” parameters, which may come to a different conclusion or view than before.

### 4.5. Interaction with Composites Factors

As the characteristics of a pure polymer may not satisfy requirements sometimes, people turn their attention to FDM-based composite materials [145], such as polymer matrix composites [146,147], bio-composites [148,149], nanocomposites [150,151], and fiber-reinforced composites [152,153], which have advantages of high mechanical performance and multi-function. However, the intrinsic properties of different composite materials, such as flow and fiber orientation, solidification behavior, and deformation [142], make it difficult for process parameters optimization related to composite materials. For example, Camineroa et al. [154] examined the influence of fiber volume, layer thickness, and build orientation on the impact properties of continuous fiber-reinforced composites. They noted that the interaction between fiber orientation and build orientation significantly led to different impact strengths for on-edge and flat specimens. In the study of Osman and Atia [155], a significant reduction of tensile modulus was observed for specimens with 45° raster angle, with the increase of rice straw content in the ABS-rice straw composite material. However, this phenomenon was insignificant overall for specimens with a 0° raster angle. In a word, the complicated influences of process parameters on the properties of composites, which are coupled with material factors, remains a big challenge for future research. 

## 5. Summary, Recommendations, and Perspectives

In summary, the research of FDM process parameters is critical for improving the characteristics and quality of parts. Different process parameters may have similar or opposite influences on the mechanical properties and behavior of components, which are also affected by other factors such as materials, printers, experiments, etc. Therefore, a compressive investigation of various process parameters is necessary and helpful. Despite existing research gaps, the future of research on FDM process parameters is the most appealing, and a number of innovative explorations await newcomers in the field. The following contents, though certainly not comprehensive, point out some potential future directions and areas that require attention from the field.

### 5.1. Condition of Printing

FDM parts are printed in diverse conditions, which inevitably affect the mechanical characteristics of printed samples. The function of environmental parameters such as platform temperature [117], envelope temperature [115,156], humidity [157,158], and oxygen [159]; other print parameters such as nozzle parameter [92] and filament diameter [160], on mechanical behavior has been more or less studied, although not very extensive. In addition, how these factors impact process parameters remains a challenge and only attracts a few researchers’ attention. For example, Mohd et al. [161] found that the diameter of the ABS filament increased as it was exposed to prolonged moisture with a certain absorption rate. However, this physical change would not cause nozzle clogging, which would directly affect the print speed. The influence of the FDM process condition could be a potential future research direction in this field.

### 5.2. Experimental Standard

The current approach to mechanical testing mainly refers to the relevant standards of raw materials and formed parts in their original application fields and utilizes existing standards. There are no specific guidelines for FDM process that prescribe the method of testing mechanical properties. This is one of the reasons that variety can be found when comparing experimental results from different authors. In the existing research, two standards are widely adopted: ASTM and ISO [2,162]. However, some of the standards are intended for materials containing high modulus fibers and are not directly applicable to samples made with FDM process. On the other hand, studies have shown certain composite standards actually improve test consistency on FDM materials [163]. Therefore, a suite of standard test methods should be developed to measure the mechanical property of parts by the FDM process. The authors hope researchers in related fields can work together to solve this urgent and important problem.

### 5.3. Multi-Parameters Optimization

The properties of FDM built parts exhibit high dependence on process parameters and can be improved by setting parameters at suitable levels. Consequently, experimental approaches are usually adopted to obtain the optimal combination, including Taguchi design [164,165], fractional factorial design [166,167], full factorial design [168,169], face-centered central composites design (FCCCD) [26,79], along with analysis methods such as analysis of variance (ANOVA) [165,166] or signal-to-noise ratio (S/N) [164,170]. Furthermore, some researchers establish the mathematical model between response and parameters (e.g., response surface methodology (RSM) [171,172]) and optimize with various algorithms. For example, particle swarm optimization (PSO) [172,173], artificial neural network (ANN) [134], bacterial foraging optimization(BFO) [26], genetic algorithm (GA) [174], surrogate-based optimization [175], naked mole-rat algorithm (NMRA) [176], and other heuristic optimization methods [177].

Although these optimization methods have achieved satisfactory results, their applicability is limited to some specific problems. In addition, the optimal result may not be achievable in practice, restrained by the parameters setting of the FDM machine. Therefore, exploring new optimization strategies with high efficiency and broad applicability is an attractive prospect. Besides, multi-objective optimization is a more challenging and complex topic [132,165,178], since the optimal result may correspond to multiple parameter combinations. Therefore, there is a need for more research efforts on multi-parameters optimization for the FDM process in the future.

### 5.4. Post-Processing Technique

Many studies have verified that there are some shortcomings in FDM components that cannot be overcome by only optimizing process parameters. These shortcomings, such as shape distortion, microvoids, uneven fiber distribution, and stairs-stepping effect [179], directly affect the mechanical characteristics of FDM parts. Therefore, post-processing techniques [180], including chemical treatment [181,182,183], heat treatment [184,185,186], laser treatment [187,188], and ultrasound treatment [189,190], are often adopted to improve mechanical strength and print quality of parts. However, these treatments may have influences on structural performance as well as process parameters. For instance, heat treatment can enhance the mechanical strength of printed products by improving crystallinity and removing residual stress of polymers [191]. At the same time, this treatment can result in changes in porosity due to annealing temperature as well, which will affect the infill density consequently [185]. Another example is ultrasound treatment. Mohamed et al. [192] used an ultrasonic transducer to improve the surface quality of components with different frequencies, and they observed from the result that the surface roughness was significantly smoother than before, together with a decrease in road width and layer thickness. Therefore, the optimal values obtained from process parameter optimization (classified as pre-processing) may change after post-processing, which needs to be paid more attention to.

### 5.5. Facing Real Parts

Most studies in the literature focus on “dog bone” samples to analyze the function of process parameters. It should be noted that the conclusion or result obtained from “lab experiment” may not apply to real applications. The review shows that there are only a couple of reports on improving the mechanical performance of a real part. For example, Zaman et al. [128] optimized five process parameters on compressive strength of drilling grid from the aerospace industry using the Taguchi design of experiments. Lee et al. [164] analyzed the relationship between process parameters and elastic performance of a compliant catapult using the Taguchi method. The maximum throwing distance was achieved by setting optimal parameters combination obtained. Since FDM products are ultimately used in practical applications, more research on real objects needs to be carried out, which can be another direction for future research.

### 5.6. Combination with 4D Printing

4D printed structures can change shape or property by stimulus, showing innovation and smartness, which has attracted unprecedented interest in recent years [193]. With the increasing application of FDM printers for 4D printing, the effect of process parameters on shape memory effect (SME) for smart materials is becoming a research hotspot [194]. For example, Kačergis et al. [105] evaluated the impact of platform temperature, print speed, and number of layers on the behavior of shape-shifting ‘hinge’ structure. They pointed out the higher print speed and lower platform temperature resulted in a higher deformation angle. In addition, the more active layers, the more time for shape recovery. Rajkumar and Shanmugam [195] analyzed the mechanisms of process parameters, such as infill density, thickness, and print speed, on shape-transformation, based on which they applied the results in manufacturing controllable curved components. In fact, there exist many unknown problems for 4D printing to be investigated, such as material behaviors, shape-shifting effects, and actuation methods [196] for smart and multi-materials obtained through the FDM approach. Therefore, research on the application of the FDM technique in printing 4D structures is exciting and appealing work awaiting further exploration.

## Figures and Tables

**Table 1:** Build direction.

Study	Process Parameters	Mechanical Properties	Materials	Machines
Ashtankar et al. [25]	Build orientation	Tensile strength, compressive strength	ABS	Dimension BST
Lee et al. [29]	Build orientation	Compressive strength	ABS	MIT 3D Printer
Gorski et al. [30]	Build orientation	Tensile strength	ABS	Dimension BST 1200
Hernandez et al. [31]	Build orientation	Compressive strength, tensile strength, flexural strength	ABS	uPrint SE Plus
Zou et al. [33]	Build orientation	Tensile strength, Young’s modulus, Poisson’s ratio	ABS	Dimension SST 1200 es
Domingo-Espin et al. [35]	Build orientation	Tensile strength, stiffness	PC	Stratasys Fortus 400 mc
Smith and Dean [36]	Build orientation	Tensile strength, modulus	PC	Stratasys Vantage SE
Bagsik et al. [79]	Build orientation	Tensile strength, compressive strength	PEI	Stratasys Fonus 400 mc
Upadhyay et al. [119]	Build orientation	Tensile strength, compressive strength	ABS P400	FDM SST-768
Rohde et al. [12]	Build orientation, raster angle	Shear strength	ABS, PC	Stratasys Fortus 360 mc, Ultimaker 2
Durgun and Ertan [23]	Build orientation, raster angle	Tensile strength, flexural strength.	ABS P430	Dimension BST
Rodriguez et al. [24]	Build orientation, raster angle	Strength, stiffness	ABS	
Bertoldi et al. [32]	Build orientation, raster angle	Tensile strength, modulus, Poisson’s ratio,	ABS	Stratasys FDM 1650
Zaldivar et al. [37]	Build orientation, raster angle	Tensile strength, failure strain, modulus, Poisson’s ratio, thermal, expansion coefficient	PEI	Stratasys Fortus 400 mc
Taylor et al. [38]	Build orientation, raster angle	Flexural strength	PEI	Stratasys Fortus 400 mc
Bellini and Güçeri [55]	Build orientation, raster angle	Tensile strength, flexural strength	ABS	Stratasys FDM 1650
Balderrama-Armendariz et al. [56]	Build orientation, raster angle	Ultimate shear strength, 0.2%yield strength, shear modulus, fracture strain	ABS	Stratasys Fortus 400 mc
Cantrell et al. [57]	Build orientation, raster angle	Tensile strength, failure strength, Poisson’s ratio, modulus	ABS, PC	Stratasys Fortus 360 mc, Ultimaker 2
Raney et al. [34]	Build orientation, infill density	Tensile strength, flexural strength	ABS	uPrint SE Plus
Torrado and Roberson [58]	Build orientation, raster pattern	Tensile strength, anisotropic property	ABS	Lulzbot TAZ 4
Wang et al. [7]	Build direction, layer thickness, deposition style	Tensile strength	ABS P400	Dimension BST
Kamaal et al. [120]	Build direction, infill density, layer thickness	Tensile strength, impact strength	CF/PLA composite	Ypanx Falcon
Tanikella et al. [121]	Building orientation, mass, color	Tensile strength	Ninjaflex, SemiFlex, HIPS, TGLase, Nylon, ABS, PC	Lulzbot TAZ 3.1 and 4

**Table 2:** Raster angle.

Study	Process Parameters	Mechanical Properties	Materials	Machines
Es-said et al. [40]	Raster angle	Tensile strength, modulus of rupture, impact resistance	ABS P400	Stratasys FDM 1650
Ahn et al. [44]	Raster angle	Tensile strength	ABS	
Magalhães et al. [45]	Raster angle	Tensile strength, Young’s modulus,	ABS P400	Stratasys FDM 2000
Ziemian et al. [46]	Raster angle	Tensile strength, fatigue strength	ABS	Stratasys Vantage-i
Garg et al. [48]	Raster angle	Tensile strength, flexural strength	ABS P400	Stratasys Mojo
Ziemian et al. [49]	Raster angle	Tensile strength, compressive strength, flexural strength, impact strength, fatigue property	ABS	Stratasys Vantage-i
Hart and Wetzel [53]	Raster angle	Fracture property	ABS M30	Lulzbot Taz 6
Arbeiter et al. [54]	Raster angle	Fracture property	PLA	Hage 3DpA2
Carneiro et al. [68]	Raster angle	Tensile strength	PP, Glass/PP composite	Prusa i3
Liu et al. [122]	Raster angle	Tensile property, flexural property	PLA/SCB composite	S1 Architect 3D
Letcher et al. [123]	Raster angle	Tensile strength, flexural strength, fracture property	PLA	MakerBot Replicator 2x
Zhou et al. [47]	Raster angle, layer thickness	Tensile strength	PP/PC composite	LeistritzZSE 18 HPe
Diaconescu et al. [52]	Raster angle, layer thickness	Tensile strength	ABS	MakerBot 2X
Letcher et al. [59]	Raster angle, number of layers	Tensile strength, modulus of elasticity	ABS	MakerBot Replicator 2x
Kung et al. [109]	Raster angle, number of contours, specimen size	Tensile strength	PLA	RepRap 3D printer

**Table 3:** Layer thickness.

Study	Process Parameters	Mechanical Properties	Materials	Machines
Vaezi and Chua [43]	Layer thickness	Tensile strength, flexural strength	ZP102	Z510/Cx printer
D’Amico et al. [70]	Layer thickness	Tensile strength, flexural strength	ABS	Makerbot 2X
Ayrilmis et al. [124]	Layer thickness	Tensile strength, flexural strength	PLA/wood composite	Zaxe 3D printer
Somireddy et al. [42]	Layer thickness, raster angle	Flexural property	ABS-P430	Stratasys μ printer
Rankouhi et al. [62]	Layer thickness, raster angle	Tensile strength, elastic modulus	ABS	Makerbot Replicator 2x
Wu et al. [65]	Layer thickness, raster angle	Tensile strength, compressive strength, flexural strength	PEEK, ABS P430	Custom-built printer
Garg and Bhattacharyab [66]	Layer thickness, raster angle	Tensile strength	ABS	uPrint SE, Plus and Mojo printers
Knoop et al. [125]	Layer thickness, build orientation	Tensile strength, compressive strength, flexural strength	Nylon	Stratasys Fortus 400 mc
Chacon et al. [27]	Layer thickness, build orientation, print speed	Tensile strength, flexural strength, stiffness	PLA	WitBox desktop 3D printer
Uddin et al. [39]	Layer thickness, build orientation, raster angle	Young’s modulus, yield strength, failure strength	ABS	Zortrax M200
Tymrak et al. [63]	Layer thickness, raster angle, color	Tensile strength, elastic modulus	ABS, PLA	A series of open-source3D printers
Dong et al. [69]	Layer thickness, number of layers, infill density	Tensile strength, flexural strength, impact strength	PLA/wood composite	MakerBot Replicator 2x

**Table 4:** Infill density.

Study	Process Parameters	Mechanical Properties	Materials	Machines
Alvarez et al. [90]	Infill density	Tensile strength, impact resistance	ABS	Makerbot Replicator 2x
Martikka et al. [91]	Infill density	Tensile properties, impact strength	PLA/wood composite	Profi3Dmaker
Aw et al. [93]	Infill density, infill pattern	Tensile property	CABS/ZnO composite	RepRap Mendelmax 1.5
Fernandez-Vicente et al. [98]	Infill density, infill pattern	Tensile strength, Young’s modulus	ABS	RepRap Prusa i3
Kerekes et al. [94]	Infill density, layer thickness	Tensile property	ABS-M30	Stratasys uPrint SE Plus
Lužanin et al. [95]	Infill density, layer thickness, raster angle	Flexural strength	PLA	Makerbot Replicator 2
Gomez-Gras et al. [92]	Infill density, layer thickness, nozzle diameter, print speed	Fatigue performance	PLA	Prusa i3
Griffithsa et al. [112]	Infill density, building direction, number of contours, layer thickness	Tensile strength, Young’s modulus	PLA	Makerbot Replicator 2

**Table 5:** Infill pattern.

Study	Process Parameters	Mechanical Properties	Materials	Machines
Ebel et al. [126]	Infill pattern	Tensile strength	PLA, ABS	CB printer, Felix 1.0e
Baich et al. [9]	Infill pattern, infill density	Tensile strength, compressive strength, flexural strength	ABS P430	Stratasys Fortus 200 mc
Cho et al. [96]	Infill pattern, layer thickness	Tensile strength, modulus, yield stress	PLA	
Akhoundi et al. [99]	Infill pattern, infill density	Tensile strength, flexural strength, modulus	PLA	Laboratory FDM 3D printer
Dave et al. [97]	Infill pattern, build orientation, infill density	Tensile strength	PLA	Open-source FDM printer
Vinoth Babu et al. [127]	Infill pattern, layer thickness, infill density	Tensile property, flexural property	CF/PLA composite	Raise 3D V2 N2 Hot end
Zaman et al. [128]	Infill pattern, layer thickness, number of contours, infill density	Compressive strength	PLA, PETG	Makerbot Replicator 2X, Open Edge HDE printer
Nagendra and Prasad [100]	Infill pattern, layer thickness, extrusion temperature, raster angle, infill density	Tensile strength, flexural strength, impact strength, compressive strength	Nylon/Aramid composite	

**Table 6:** Air gap.

Study	Process Parameters	Mechanical Properties	Materials	Machines
Rodriguez et al. [71]	Air gap	Tensile strength, stiffness	ABS P400	Stratasys FDM1600
Too et al. [72]	Air gap	Compressive strength, porosity	ABS P400	Stratasys FDM1650
Dawoud et al. [73]	Air gap, raster angle	Tensile strength, flexural strength, impact strength	ABS	DIY FDM machine
Masood et al. [74]	Air gap, raster width, raster angle	Tensile strength	PC	Stratasys Vantage
Hossain et al. [76,77]	Air gap, raster angle, contour width, raster width	Tensile strength	PC	Stratasys Fortus 900 mc
Montero et al. [78]	Air gap, raster angle, raster width, extrusion temperature, color	Tensile strength	ABS P400	Stratasys FDM 1650
Bagsik and Schöppner [79]	Air gap, build orientation, raster angle, raster width	Tensile strength	PEI	Stratasys Fortus 400 mc
Ang et al. [83]	Air gap, raster width, build orientation, build layer, build profile	Compressive strength, porosity	ABS	Stratasys FDM 1650

**Table 7:** Print speed.

Study	Process Parameters	Mechanical Properties	Materials	Machines
Christiyana et al. [103]	Print speed, layer thickness	Tensile strength, flexural strength	ABS/ hydrous magnesium silicate composite	3D protomaker STURDY
Santana et al. [104]	Print speed, extrusion temperature	Flexural strength	PLA	IFSC 3D printer
Li et al. [21]	Print speed, layer thickness, air gap	Tensile strength	PLA	MakerBot Z18
Kačergis et al. [105]	Print speed, number of layers, platform temperature	Deformation	PLA, TPU	Anycubic Prusa i3
Attoye et al. [129]	Print speed, build orientation, extrusion temperature	Young’s modulus, yield strength	PLA, ABS	MakerBot
Ning et al. [101]	Print speed, raster angle, extrusion temperature, layer thickness	Tensile strength, Young’s modulus, yield strength	CFRP composite	Creatr AM machine

**Table 8:** Number of contours.

Study	Process Parameters	Mechanical Properties	Materials	Machines
Croccolo et al. [111]	Number of contours, build orientation	Tensile strength, stiffness	ABS-M30	
Lanzotti et al. [61]	Number of contours, layer thickness, raster angle	Tensile strength	PLA	Reprap Prusa I3
Mahmood et al. [110]	Number of contours, infill density, cross-sectional area	Tensile strength	ABS	Makerbot Replicator 2X
Chokshi et al. [130]	Number of contours, layer thickness, infill pattern	Tensile strength, flexural strength	PLA	Prusa MK3S
Gebisa and Lemu [80]	Number of contours, air gap, raster width, raster angle, contour width	Flexural property	PEI	Stratasys Fortus 450
Torres et al. [131]	Number of contours, extrusion temperature, print speed, raster angle, infill density, layer thickness	Tensile strength, fracture property	PLA	MakerBot Replicator2

**Table 9:** Extrusion temperature.

Study	Process Parameters	Mechanical Properties	Materials	Machines
Aliheidari et al. [113]	Extrusion temperatures	Fracture property	ABS	Felix pro I printer
Sun et al. [117]	Extrusion temperature	Flexural strength	ABS P400	Stratasys FDM 2000
Yang [118]	Extrusion temperature	Tensile property, flexural property, compressive strength	PLA/wood composite	Creator Pro
Rinanto et al. [114]	Extrusion temperature, infill density, raster angle	Tensile strength	PLA	Politeknik ATMI Surakarta FDM Machine
Sun et al. [115]	Extrusion temperature, infill density	Tensile strength	PEEK	High temperature FDM type 3D printer
Abouelmajd et al. [132]	Extrusion temperature, print speed, raster angle	Flexural strength, stiffness	PLA	WANHAO Duplicator 4S
Deng et al. [82]	Extrusion temperature print speed, layer thickness, infill density	Tensile strength, flexural strength impact strength	PEEK	Custom-built FDM equipment
Leite et al. [116]	Extrusion temperature, infill density, raster orientation, layer thickness	Tensile strength, yield strength, modulus of elasticity, elongation at break	PLA	Ultimaker 2 machine

**Table 10:** Raster width.

Study	Process Parameters	Mechanical Properties	Materials	Machines
Gkartzou et al. [87]	Raster width	Tensile strength, Young’s modulus	PLA/ lignin composite	Zmorph 2.0 S
Rajpurohit and Dave [64]	Raster width, layer thickness, raster angle	Tensile property	PLA	Open-source FDM printer
Slonov et al. [75]	Raster width, air gap, raster angle,	Tensile strength, elastic modulus, impact strength	PPSF	Stratasys Fortus 400 mc
Rajpurohit and Dave [89]	Raster width, layer thickness, raster angle	Flexural property	PLA	Open-source FDM printer

**Table 11:** Others.

Study	Process Parameters	Mechanical Properties	Materials	Machines
Toro et al. [13]	Layer thickness, raster angle, infill pattern, infill density.	Tensile strength, flexural strength	CRF/Nylon composite	Ultimaker 2 Extended +.
Rayegani and Onwubolu [84]	Build orientation, raster angle, raster width, air gap	Tensile strength	ABS	Stratasys Fortus 400 mc
Panda et al. [133]	Layer thickness, raster angle, raster width, air gap	Tensile strength	ABS P400	Fortus 400 mc
Sood et al. [22]	Layer thickness, build orientation, raster angle, raster width, air gap	Tensile strength, flexural strength, impact strength	ABS P400	FDM Vantage SE machine
Panda et al. [26]	Layer thickness, build orientation, raster angle, raster width, air gap	Tensile strength, flexural strength, impact strength	ABS P400	FDM Vantage SE machine
Onwubolu and Rayegani [85]	Layer thickness, build orientation, raster angle, raster width, air gap	Tensile strength	ABS P400	FDM 400 mc machine
Liu et al. [86]	Layer thickness, build orientation, raster angle, raster width, air gap	Tensile strength, flexural strength, impact strength	PLA	MakerBot Replicator2
Giri et al. [134]	Air gap, raster width, layer thickness, build orientation, raster angle, number of contours	Tensile strength	PLA	Customized printer

## Data Availability

Not applicable.
